# Supplementary material for: A new type of radial basis functions for problems governed by partial differential equations
Source: PLoS One. 2023 Nov 29;18(11):e0294938. doi: 10.1371/journal.pone.0294938 (PMC10686449; doi:10.1371/journal.pone.0294938)
Supplement: Attachment — Submitted filename: PLOS ONE.docx [file pone.0294938.s001.docx]

The authors are invited to address the following minor comments :

1- Add SI units in the nomenclature table.
2- The motivation part of the introduction should be improved with a clear specification of the objectives and novelties.
3- Organize the sections of the paper more personally.
4- Provide suitable references for all equations.

5-Enhance the introduction by citing the following references:

*J. A. C.Weideman and S. C. Reddy, “A matlab differentiation matrix suite,” ACM Transactions on Mathematical Software 26, 465–519 (2000).

*E. Beutler, “Spectral methods in matlab,” (SIAM, Philadelphia, 2000).

6-As for the conclusion, it should be significantly expanded, and all the main aspects should be covered in	detail.
