# Supplementary material for: A new type of radial basis functions for problems governed by partial differential equations
Source: PLoS One. 2023 Nov 29;18(11):e0294938. doi: 10.1371/journal.pone.0294938 (PMC10686449; doi:10.1371/journal.pone.0294938)
Supplement: Attachment — Submitted filename: Reply to Reviewer 2.docx [file pone.0294938.s002.docx]

**Reply to Reviewer 2**

1- Add SI units in the nomenclature table.

Since the current research focuses on an numerical investigation, it is not based on a precise background of the related nomenclature. All the nomenclatures are mathematical symbols, so SI units are not applicable.

2- The motivation part of the introduction should be improved with a clear specification of the objectives and novelties.

The motivation part of the introduction is improved in the revised manuscript. A specification of the objectives and novelties are presented in the introduction.

3- Organize the sections of the paper more personally.

We have organized sections of the paper under the suggestion of the journal.

4- Provide suitable references for all equations.

References for all equations are checked carefully.

5- Enhance the introduction by citing the following references:

*J. A. C. Weideman and S. C. Reddy, “A matlab differentiation matrix suite,” ACM Transactions on Mathematical Software 26, 465–519 (2000).

*E. Beutler, “Spectral methods in matlab,” (SIAM, Philadelphia, 2000).

These excerllent references are added in the introduction.

6- As for the conclusion, it should be significantly expanded, and all the main aspects should be covered in	detail.

We have expanded the conclusion in the revised manuscript.
